# State of Art of Dose Individualization to Support tacrolimus drug monitoring: What’s Next?

**DOI:** 10.3389/ti.2025.14201

**Published:** 2025-09-01

**Authors:** N. Lloberas, B. Fernández-Alarcón, A. Vidal-Alabró, H. Colom

**Affiliations:** ^1^ Nephrology Department, Hospital Universitari de Bellvitge-Institut d'Investigació Biomèdica de Bellvitge (IDIBELL), Barcelona, Spain; ^2^ Biopharmaceutics and Pharmacokinetics Unit, Department of Pharmacy and Pharmaceutical Technology and Physical Chemistry, School of Pharmacy and Food Sciences, University of Barcelona, Barcelona, Spain

**Keywords:** kidney transplantation, pharmacogenetics, pharmacokinetics, population pharmacokinetics, tacrolimus, mathematical modeling

## Abstract

Tacrolimus is an immunosuppressant with a narrow therapeutic index and a high intra- and inter-patient variability showing significant challenges in optimal dosing and monitoring. Historically, pre-dose concentration monitoring and simplified area under the curve measurements have been the standard approach. However, recent advances in pharmacokinetic modeling have improved individualized dosing strategies, moving beyond empirical methods. This review explores the evolving landscape of Tacrolimus therapeutic drug monitoring, focusing on advanced modeling techniques that support personalized dosing. Key methodological approaches include Population Pharmacokinetic (PopPK) modeling, Bayesian prediction, Physiologically-Based Pharmacokinetic (PBPK) modeling, and emerging machine learning and artificial intelligence technologies. While no single method provides a perfect solution, these approaches are complementary and offer increasingly sophisticated tools for dose individualization. The review critically examines the potential and limitations of current modeling strategies, highlighting the complexity of translating advanced statistical and mathematical techniques into clinically accessible tools. A significant challenge remains the gap between sophisticated modeling techniques and the practical usability for healthcare professionals. The need for user-friendly platforms is emphasized, with recognition of existing commercial solutions while also noting their inherent limitations. Future directions point towards more integrated, intelligent systems that can bridge the current technological and practical gaps in personalized immunosuppressant therapy.

## Introduction

The landscape of solid organ transplantation witnessed a transformative shift during the 1990s, with the new immunosuppressive strategies significantly changing short-term graft and patient survival [1]. Despite these advances, long-term outcomes continue to front challenges, with tacrolimus remaining the cornerstone of post-transplant immunosuppression [1]. Tacrolimus pharmacokinetic is characterized by a narrow therapeutic window and high variability between and within patients underscoring the critical importance of personalized therapeutic drug monitoring (TDM). Transplant medicine represents a delicate balance between immunological management and pharmacological precision. The current clinical paradigm presents a critical challenge: preventing organ rejection while simultaneously avoiding the risks of over-immunosuppression. Current standard practices, particularly weight-based dosing, are a poor predictor of tacrolimus exposure, with only 20%–35% of transplant recipients achieving target therapeutic levels at first steady state [2–5]. During this period, accurate adjustments of immunosuppressants are vital to prevent risks such as allograft rejection, nephrotoxicity, and therapeutic failure [6–10].

Several studies have demonstrated that tacrolimus levels below the therapeutic target are associated with an increased risk of allograft rejection within the first 3–6 months post-transplantation [11, 12]. Careful management of immunosuppression is crucial, as under-immunosuppression can lead to acute rejection, while over-immunosuppression increases the risks of infections and malignancies. As transplant patient life expectancy continues to improve, the focus has evolved from preventing early graft rejection to managing the long-term consequences of prolonged immunosuppressive therapy and its associated adverse effects [13]. Adjusting both under- and over-exposure remains a significant challenge due to the considerable variability among transplant recipients [14, 15].

Population pharmacokinetic (PopPK) modeling has emerged as a promising bridge research insights and clinical application, offering a sophisticated approach for drug dosing that incorporates multiple variables affecting drug metabolism and distribution. The integration of single Nucleotide polymorphisms (SNP), particularly CYP3A variants, provides opportunities for more precise dosing strategies. Guidelines from both the Clinical Pharmacogenetic Implementation Consortium (CPIC), the Dutch Pharmacogenomics Working Group, and the International Association of Therapeutic Drug Monitoring and Clinical Toxicology (IATDMCT) have emphasized the importance of genetic variants in tacrolimus metabolism. However, a significant gap remains between these theoretical frameworks and their practical implementation in clinical care.

Current TDM approaches rely on a trial-and-error method that can take up to 3 weeks to achieve target drug levels, leaving patients vulnerable to potential complications. Recent modeling advances have expanded the variables considered in tacrolimus pharmacokinetics, including clinical factors such as age, body composition, albumin levels, demographic characteristics like ethnicity, and SNPs affecting drug transport and metabolism. The concentration-to-dose (C/D) ratio has emerged as a valuable tool for ongoing dose adjustment [16–18], while Bayesian modeling approaches show promise for more precise initial dosing strategies.

This review aims to explore the complex landscape of tacrolimus pharmacokinetic variability by critically analyzing PopPK models and advanced modeling strategies. These include Bayesian prediction, Physiologically-Based Pharmacokinetic (PBPK) modeling, and machine learning technologies as innovative tools for individualizing immunosuppressive therapy. The authors seek to bridge sophisticated mathematical techniques with clinical implementation, highlighting the need for user-friendly platforms that can translate complex statistical methodologies into accessible clinical tools for therapeutic optimization.

## Conventional Therapeutic Drug Monitoring of Tacrolimus

Traditional TDM protocols for tacrolimus starting dose fail to account for the multifaceted nature of tacrolimus pharmacokinetics. This conventional approach runs under the presumption that a linear relationship exists between body weight and both drug clearance and volume of distribution–an assumption that has proven to not be the best tool to apply in clinical practice [15]. Tacrolimus maintenance dosing is usually adjusted based on pre-dose trough levels (C0), a widely accepted parameter for TDM due to its presumed strong correlation with the area under the curve (AUC) [19].

### Pre-Dose Concentration Versus AUC

The measurement of C0 has emerged as the standard of care in transplant centers globally. However, the correlation between C0 and AUC has shown varying degrees of reliability across different studies [19]. Recent real-world data analysis of patients in their second and third post-transplant years demonstrated that while both C0 and AUC correlated with BPAR incidence, AUC proved superior in identifying patients with exposure irregularities despite apparently adequate C0 levels [20]. The C0/dose ratio has emerged as a valuable predictor of CNI nephrotoxicity, with studies by Thölking et al. [16, 21, 22] and others [23, 24] demonstrating its prognostic value for renal function outcomes. Fast metabolizers, identified by lower C0/dose ratios, showed higher peak concentrations despite similar trough levels, suggesting that C0 monitoring alone might miss important exposure patterns [17, 25].

### Sources of Variability in Tacrolimus Pharmacokinetics

Numerous factors have been identified that impact tacrolimus pharmacokinetics, contributing to the inter-patient variability [26–28]. Tacrolimus displays variable absorption in the gastrointestinal tract, with factors like gastric pH, motility, and the presence of food impacting its bioavailability. Reduced absorption can be observed in conditions such as delayed gastric emptying or gastrointestinal inflammation, leading to subtherapeutic drug levels. Gastrointestinal motility disorders, particularly diarrhea, can markedly enhance tacrolimus absorption, potentially leading to toxic levels in certain patients [29–33]. Lemahieu et al mentioned a decreased intestinal p-glycoprotein activity as a potential cause for higher absorption of tacrolimus. Moreover, the accelerated movement through the intestinal tract results in increased tacrolimus exposure to both the distal portion of the small intestine and colonic tissue, where absorption can occur [34]. This drug is extensively metabolized in the liver by cytochrome P450 enzymes, primarily CYP3A4 and CYP3A5, with hepatic function variations significantly altering drug clearance.

Pharmacokinetic variability is further complicated by physiological factors like erythrocyte binding, where lower hematocrit levels result in higher free drug concentrations and increased clearance [27]. Alterations in albumin levels and hematocrit enhance tacrolimus elimination and dosing requirements, although these changes do not substantially impact the unbound drug fraction [27, 35–41].

Patient demographics play a crucial role, with pediatric patients requiring higher doses due to enhanced hepatic enzyme activity, while elderly individuals (≥65 years) experience slower metabolism from age-related liver and kidney function decline, potentially leading to up to 50% higher tacrolimus exposure despite lower dose-to-body weight ratios [42–47].

Drug metabolism through oxidative pathways predominantly involves the Cytochrome P450 (CYP) 3A subfamily, which significantly influences tacrolimus concentrations [44, 45]. CYP3A5*1 (*1 allele expressers) (rs776746) demonstrate markedly increased tacrolimus clearance, requiring approximately 50% higher doses to achieve therapeutic levels compared to non-expressors (*3/*3 genotype) [50–54]. This pharmacogenetic effect underscores the importance of CYP3A5 genotyping in optimizing tacrolimus therapy [2, 50–52, 55]. In contrast, the CYP3A4*22 variant also demonstrates clinical relevance. Carriers of the T variant allele exhibit reduced CYP3A4 activity [56], requiring approximately 33% lower tacrolimus doses [57]. The combined influence of CYP3A4/5 SNPs according to metabolizer phenotypes have significant impact on tacrolimus pharmacokinetic. Different studies have demonstrated that integrating both CYP3A5/4 genotypes can explain over 60% of observed variability in tacrolimus concentrations [57, 58]. Current clinical guidelines from CPIC and IATDMCT [15] recommend increasing doses by 1.5–2 times for patients with enhanced metabolism, highlighting the practical application of this genetic information in personalizing tacrolimus therapy.

Tacrolimus transport is primarily mediated by P-glycoprotein (Pgp), an efflux pump encoded by the ABCB1 gene, which facilitates drug movement across multiple physiological barriers including intestinal epithelium, hepatic tissue, blood-brain barrier, renal tubules, pancreatic cells, and lymphocytic membranes [59]. The ABCB1 gene’s widespread distribution is crucial in determining tacrolimus pharmacokinetics, particularly in absorption, distribution, and elimination [49]. Over 50 ABCB1 SNPs have identified with three key variants in clinical research: 3435C>T (rs1045642), 1236C>T (rs1128503), and 2677G>T/A (rs2032582). These SNPs exist in linkage disequilibrium, suggesting potential coordinated effects on Pgp function. However, despite theoretical expectations of decreased Pgp activity associated with these variants, multiple clinical investigations have failed to demonstrate consistent correlations between these polymorphisms and systemic tacrolimus concentrations [60–65].

Drug-drug interactions with tacrolimus, primarily mediated by CYP3A4 and Pgp, are well-documented [66]. Co-administration of drugs that interact with ABCB1 and/or CYP3A can significantly alter the bioavailability and metabolism of tacrolimus [67]. This may result in high levels of immunosuppression, increasing the risk of toxicity, or in levels that are too low, raising the likelihood of organ rejection [68]. Inhibitors like azole antifungals, calcium channel blockers (e.g., verapamil, diltiazem), HIV protease inhibitors (e.g., ritonavir), macrolides (excluding azithromycin), amiodarone, and nefazodone increase tacrolimus exposure. While azole antifungals are strong inhibitors of tacrolimus metabolism, others, such as azithromycin, have minimal clinical effects. In contrast, inducers like rifampicin, anticonvulsants, and corticosteroids significantly decrease tacrolimus levels. Therefore, in addition to making dosage adjustments, therapeutic drug monitoring (TDM) is essential in clinical practice for transplant patients, especially when changes to their treatment regimen are necessary.

## Overview of Pharmacokinetic Models for Tacrolimus: Population Pharmacokinetic (PopPK) Models, Physiologically-Based Pharmacokinetic (PBPK) Models, and Machine Learning (ML) Approaches

Currently, the two primary approaches for describing the pharmacokinetics of tacrolimus and predicting its concentrations in transplant patients are population pharmacokinetic (PopPK) and physiologically based pharmacokinetic (PBPK) models. Recently, a new approach, machine learning (ML), has also emerged. While PopPK and PBPK models use differential equations, ML relies on statistical relationships between variables to make predictions.

It is worth noting that PopPK and PBPK models each have unique strengths and limitations, and they are not mutually exclusive; instead, they can be used complementarily. Table 1 summarizes the main differences between these two approaches, meanwhile Table 2 summarizes the limitations of each one.

**TABLE 1:** Summary of Characteristics of each approach, Pop-PK and PBPK models.

**Feature**	**PBPK modeling**	**Pop-PK modeling**
**Methodolgy**	Mechanistic	Empirical/Statistical
**Sparse Data analysis**	Less efficient than Pop-PK	Very useful and efficient
**Extrapolation capability**	Interspecies, age, disease states	Descriptive capability. Extrapolation only within the range of variation of the identified covariates in the target population
**Drug-Drug interactions (DDI) Prediction**	Powerful	Limited
**Special Population Suitability**	High suitability for pediatric, geriatric, disease states	Aims at identifying factors of variability within a given population
**Regulatory Acceptance**	High, especially for DDIs and special populations	High, widely used for dose recommendations
**Real-World Application**	Limited as it requires detailed physiological parameters to be available	Useful for clinical PK studies and as support tool during the therapeutic drug monitoring (TDM) using bayesian prediction

**TABLE 2:** Summary of limitations of each approach, Pop-PK and PBPK models.

**PBPK modeling**	**Pop-PK modeling**
**Model complexity and computational complexity** due to the multiple interconnected compartments and the differential equations required to define the system	**Computational complexity:** Typically, mathematically less complex than PBPK because less parameters are involved, **but large datasets and complex non-linear mixed-effects models can still require long computing times**
**Requires knowledge of species-specific anatomical, physiological and biochemical parameters** such as tissue volumes, blood flow, metabolic enzyme and transporter expression and also **drug specific** such as partition coefficients Not all these parameters can be experimentally measured, and then they have to be estimated from other data	
**Variability**: Physiological parameters (Flows, Volumes…) can vary across populations or disease states, leading to uncertainty and variability	**Large population studies are required:** Pop-PK modeling aims at identifying the sources of PK variabilty to optimize the dose regimens in the target population If the range and effect of a physiological parameter observed in the target population is small, it will be misleading to identify this as an influential covariate within the study, even though the parameter may be truly influential.Therefore it requires large population studies to capture variability, but data collection limitations may restrict the range of accuracy of covariates that are physiologically meaningful to explain PK variability in the target population
**Oversimplifications under certain circumstances:** i) Lack of homogeneity within the same compartment exists (i.e., Brain) ii) Lack of PK linearity occurs iii) Changes of physiological conditions with time	**Oversimplifications of the real-world drug processes** that have an impact on model predictions
**Software Limitations:** Lack of flexibility of some platforms to handle highly complex or non-standard models, requiring modelling expertise	**There are commercially available powerful softwares but they require expertise modelers in pharmacometrics, biostatistics and non-linear mixed-effects models** which may not be available in all clinical or research settings

### Population Pharmacokinetic Models

**FIGURE 1:**
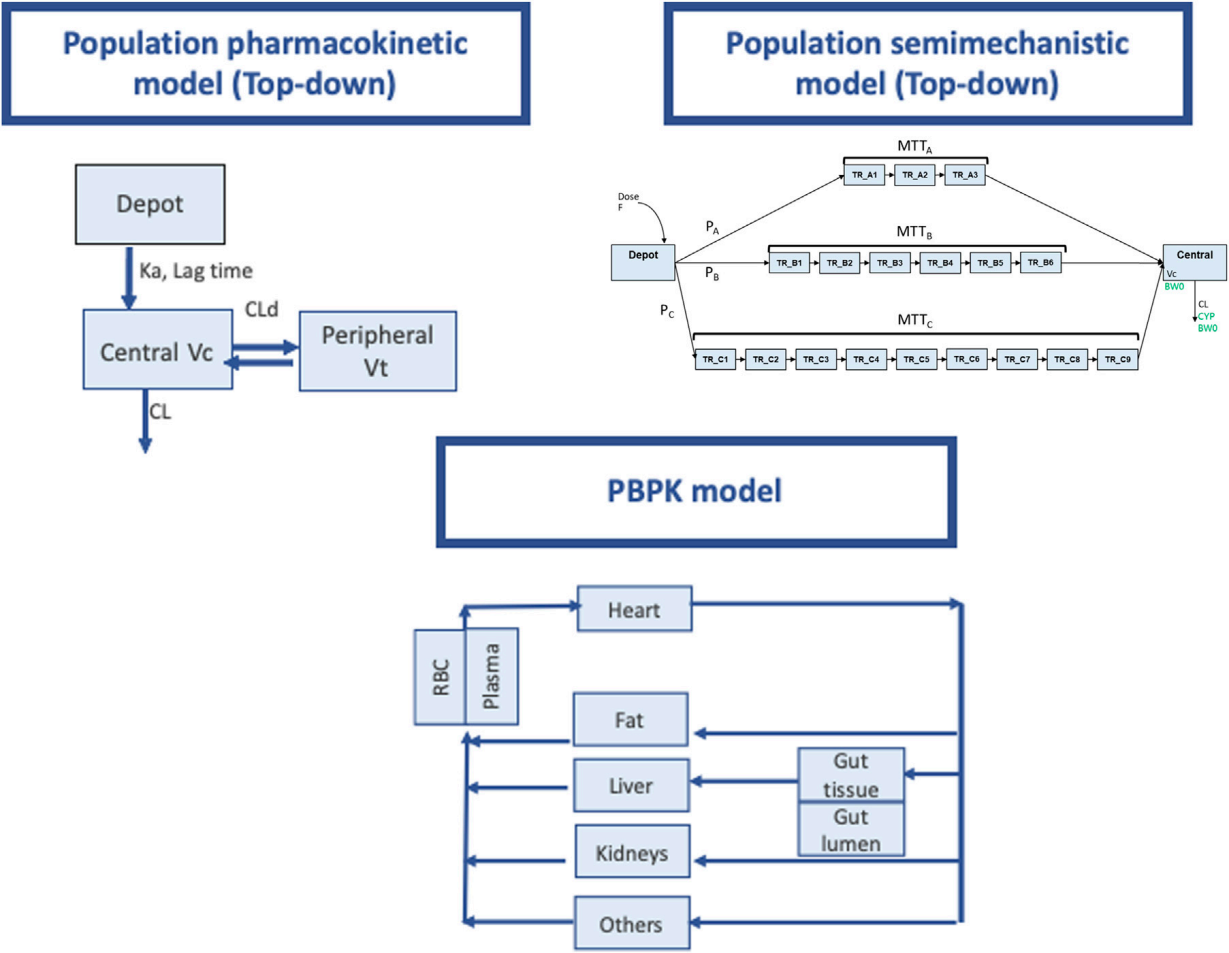
Pharmacokinetic modeling approaches used for PK prediction of tacrolimus. Upper left pannel: Schematic representation of a population pharmacokinetic model with a deport compartiment and two open compartments (central and peripheral). Ka absorption rate constnat, Vc and Vp central and peripheral distribution volumes. CLd distribution clearance, CL elimination clearance. “Central” and “peripheral” compartments, do not represent actual physiological tissues and provide only empirical descriptions of drug pharmacokinetics in the body. The model building process starts with simple models and increases in complexity depending on the complexity of the pharmacokinetic behavior of the drug under study. This approach based on observed data, is widely known as “top-down”. Upper right panel (Taken from Henin et al, with permission): Schematic representation of LCPT model structure. F relative bioavailability; P_A_, P_B_, P_C_ proportion of dose following fast (chain A), medium (chain B) and slow (chain C) absorption processes respectively; MTT_A_ mean transit time for chain A (fast absorption); MTT_B_ mean transit time for chain B (medium absorption); MTT_C_ mean transit time for chain C (slow absorption); TR_AX (X being from 1 to 3) Xth transit compartment in chain A; TR_BX (X being from 1 to 6) Xth transit compartment in chain B; TR_CX (X being from 1 to 9) Xth transit compartment in chain C; V C volume of central compartment; CL clearance; BW0 body weight at baseline (covariate on V C and CL); CYP CYP3A5 single nucleotide polymorphism (covariate on CL). Lower panel: PBPK model (adapted form Prado-velasco et al 2019 with permission). Physiological plausibility is present in this approach flow diagram for TAC PBPK model with 4 flow-limited tissues (fat, kidneys, liver and others) and 2 membrane-limited tissues (gut and blood). The blood compartment is defined through the red blood cell- plasma component. The gastric system is comprised of a gut lumen where the TAC form is liberated following a zero-order kinetic with sink condition, a one-order absorption membrane and gut tissue perfused with blood.

The PopPK approach aims to identify the sources of variability in the pharmacokinetic profile of a drug within the target population, but sufficient data are required. This is a necessary step in the successful clinical translation of any drug. The number of subjects included in the study determines the precision and clinical relevance of the effect of a covariate. PopPK models are compartmental models that describe the dose–concentration relationship from all available data by building a model with structural and statistical components that fits the data (Figure 1). PopPK modeling enables us to optimize the dose regimens, based on the predictive factors of PK variability in the target population.

#### Model-Informed Precision Dosing

Model-Informed Precision Dosing (MIPD) is an advanced quantitative approach used to optimize individualized dosing. This method combines TDM measurements with PopPK models to individualize treatment regimens by applying Bayesian forecasting [69, 70].

MIPD is a promising alternative to conventional dosing approaches. It enables faster initial dose titration through *a priori* MIPD based on baseline covariate values that predict variability. It also improves subsequent achievement of C_0_ or AUC targets via *a posteriori* MIPD based on prior pharmacokinetic assessments and updated covariate information over time [71, 72].

Widespread use of MIPD is currently limited by several challenges, including limited clinical modelling expertise, limited generalizability and harmonization of models across different patient populations, and a lack of conclusive evidence that it actually improves outcomes [73]. Despite these barriers, considerable progress has been made, providing a valuable source of evidence to support and guide future clinical pharmacometrics efforts in the context of renal transplantation [72]. As mentioned above, tacrolimus by concentration-guided dose titration has certain limitations and the MIPD represents a viable alternative to optimize the individualized dosing regimen in transplant TDM [35, 74].

#### Model-Informed Precision Dosing Modeling Software

Several software programs have been developed to enhance the prediction of patient drug concentrations and provide individualized dose recommendations to minimize PK variability. Notably, Fuchs et al., followed by Del Valle-Moreno et al., conducted extensive reviews to catalog MIPD software tools, offering detailed descriptions of their primary features. These reviews place particular emphasis on selecting the most appropriate software tools to align with specific clinical needs [70, 75].

The use of MIPD software continues to grow, driven by its precision, advancements in PopPK models, and the expanding set of drugs that can benefit from optimization. This trend reflects an increasing awareness of the importance of dose individualization for vulnerable populations, such as elderly patients, individuals with renal or hepatic impairment, pregnant women, critically ill patients, and children. Consequently, these computer programs have become indispensable tools in routine clinical practice [70, 75].

Hoffert et al. identified seven software tools currently utilized in clinical settings to guide tacrolimus dosing for renal transplant patients: Rx Studio, PrecisePK, InsightRx Nova, MwPharm, DoseMeRX, BestDose, and ISBA [76].

PrecisePK, MwPharm, DoseMeRX, and BestDose underwent prospective validation of their tacrolimus modules prior to their integration into clinical practice. Software tools designed for clinical decision-making may obtain CE marking, which signifies compliance with European Union regulations, although this certification is not mandatory. These tools serve as decision-support systems, providing dosing recommendations to clinicians, who ultimately make the final therapeutic decisions.

For tacrolimus dosing, CE marking has been obtained by PrecisePK, InsightRx Nova, MwPharm, and DoseMeRX. Additionally, some software modules offer multiple PopPK models to facilitate MIPD for renal transplant patients. However, only InsightRx Nova and MwPharm support MIPD for pediatric populations [76].

#### Population Pharmacokinetic Models for Tacrolimus

Four comprehensive reviews of tacrolimus PopPK models have been published [74, 76–78]. Brooks et al. and Kirubakaran et al. compiled detailed information on models for solid organ transplant recipients, including transplant type, formulations, sampling times, and bioanalytical methods [74, 78]. Nanga et al. proposed a meta-model applicable across different populations [77], while Hoffert et al. reviewed MIPD software modules and covariate impacts on exposure [76].

Most studies focused on the first post-transplant year, with patients on tacrolimus, mycophenolate, and corticosteroids [74, 76–78]. For kidney transplants, models primarily covered immediate-release formulations, with fewer studies on extended-release versions like Envarsus^®^ [79–82]. NONMEM was the predominant modeling software, though some studies used non-parametric approaches like Pmetrics [74, 76–78].

Two-compartment models were most common, particularly with intensive sampling data, while trough concentration studies typically used one-compartment models. Various absorption models were tested, reflecting tacrolimus’ complex absorption patterns [47]. Most models derived from White populations, potentially limiting their applicability to other ethnic groups. Hispanic patients showed 40% lower apparent clearance compared to non-Hispanic populations [83].

Key factors affecting tacrolimus clearance include CYP3A5 genotype, hematocrit, and post-transplant time [74, 77, 78]. CYP3A5*3/*3 variant carriers show lower clearance and higher dose requirements than CYP3A5 expressors [15, 84]. Studies also examined CYP3A4, ABCB1, ABCC2, and POR28 polymorphisms [79, 85–94]. Long-term administration shows decreasing dose requirements due to reduced corticosteroids, improved CYP3A5/CYP3A4 activity, and increasing hematocrit [78]. Størset et al. standardized concentrations to 45% hematocrit for better pharmacokinetic assessments [95].

Body composition significantly affects distribution volume. Fat-free mass better predicts tacrolimus clearance than total body weight, as demonstrated by Holford and Størset [35, 95]. Overweight patients risk overexposure with weight-based dosing [96]. Bio-impedance spectroscopy studies suggfance variability [97]. Model validation remains limited compared with the high rate of published models, with few studies including external cohort validation. Zhao et al carried out external evaluation of 16 models developed in kidney transplant recipients with data from 52 external patients [72]. According to the authors, the published models were unsatisfactory in prediction- and simulation-based diagnostics, thus inappropriate for direct extrapolation correspondingly. However, Bayesian forecasting could improve the predictability considerably with priors.

### Physiologically-Based Pharmacokinetic Models

PBPK models represent a significant advancement over traditional PopPK approaches in their ability to predict drug concentrations across multiple organs. These models integrate both physicochemical properties and physiological characteristics, creating a comprehensive framework based on physiologically meaningful compartments interconnected through blood circulation. The mathematical foundation relies on mass-balance differential equations that precisely define drug movement throughout the system [98].

The architecture of PBPK models demonstrates remarkable flexibility in compartment selection, adapting to specific study objectives. In tacrolimus modeling, particular emphasis is placed on pharmacokinetically significant tissues such as red blood cells, fat, liver, and intestinal tissues, while other less relevant tissues may be consolidated into broader compartments.

Three distinct approaches have emerged in PBPK modeling, each offering unique advantages. The bottom-up approach predicts pharmacokinetics by leveraging drug physicochemical characteristics and *in vitro* ADME data. This strategy proves particularly valuable when clinical data is limited, with flexibility to be adapted to different populations through physiological parameter adjustments. In contrast, the top-down approach relies heavily on clinical data for model optimization, providing high accuracy for studied populations but with limited extrapolation capabilities. The middle-out approach bridges these methodologies, combining mechanistic and clinical data to enable iterative model refinement.

Model evaluation follows rigorous criteria as outlined in regulatory frameworks [98]. These include detailed comparisons of simulations with experimental concentration-time profiles, utilizing both graphical representations and error function analyses. Models must demonstrate consistency across various scenarios, including different doses, species, populations, and similar compounds. Sensitivity analysis plays a crucial role in identifying key parameters and establishing their plausible ranges.

The importance of PBPK modeling in drug development and clinical applications has been recognized by regulatory bodies, with both the EMA and FDA issuing comprehensive guidance documents for model evaluation. These guidelines, while primarily focused on regulatory applications, provide valuable frameworks that inform broader research applications in human drug modeling.

#### Physiologically-Based Pharmacokinetic Modeling Software

Once the entire system is defined and all relevant tissue compartments are established according to the study’s objectives, the model’s equations must be coded to enable simulations or parameter estimation, depending on the study’s goals. This coding can be done using general mathematical modeling software, commonly used by engineers, or specialized PBPK modeling software. Most of these options are commercial products [99, 100]. Generally, none of these tools are particularly beginner-friendly but offer an exponential learning curve (Table 3).

**TABLE 3:** Summary of some fo the most commonly used PBPK softwares and characteristics.

	**General mathematical modelling softwares not specific to PBPK (open softwares)**	**Characteristics (model structure not defined** ***a priori*)**
	**Company**	
MATLAB, Berkeley Madonna, ModelMaker, acsIX	http://www.mathworks.com/products/matlab/, http://www.modelkinetix.com/modelmaker/, http://www.berkeleymadonna.com/, http://www.acslX.com	Very flexible but require programming skills and modelling expertise
Phys-PK	https://www.physpk.com/	Not free programme. Very flexible. Require programming skills **but it also allows interface model building**. Exponential learning curve. User-customisation management for simulation of special populations (paediatrics, geriatrics, and hepatic and renal impairment). This is achieved by adjusting physiological and pharmacokinetic parameters according to the demographic and physiological characteristics of each group. Drug-Drug interactions
	**PBPK specific softwares (Designed softwares)**	**Characteristics (Model structure typically defined** ***a priori*)**
	**Company**	Less flexible but require less mathematical modelling expertise
GastroPlus	Simulation Plus https://www.simulations-plus.com/	Exponential learning curve. Not free programme. Customised user management for simulations in pediatrics, geriatrics and pregnancy. Also focused on dissolution, formulation development and virtual bioequivalence. Advanced compartment absorption and transit (ACAT) model to predict oral bioavailability. Drug-Drug inteactions
Phoenix-WinNonlin	Certara https://www.certara.com	Not specific for PBPK modeling and simulation, but it can be also used for this purpose. Not free programme
PK-Sim and Mobi$	Open system Pharmacology https://www.open-systems-pharmacology.org/	Exponential learning curve. Free program. Customised user management for simulations in special populations (pediatrics, geriatrics and hepatic and renal impairment, pregnancy and obesity), genetic variability.Absorption compartment models GI-Sim to predict oral bioavailability. Drug-Drug inteactions
Simcyp	Certara https://www.certara.com/software/simcyp-pbpk/	Exponential learning curve. Not free programme. Customised user management for simulations in special populations (pediatrics, geriatrics hepatic and renal impairment, pregnancy and obesity), genetic variability, reduced cardiac output). Also focused on dissolution, formulation development and virtual bioequivalence, food effect. ADAM model: Advanced dissolution, absorption metabolism model, to predict oral bioavailability. Drug-Drug interactions. Mechanistic transdermal absorption model

(*) In general, all them allow the simulation of different clinical scenarios, such as dose changes, chronic administration, or enzymatic variability, which is useful for optimizing therapy and assessing possible drug-drug interactions. This table highlights key characteristics of the software solutions, including whether they are free or paid software and the specific capabilities they offer are showed.

$Mobi allows custom models using programming approaches within PK-Sim.

#### Physiologically-Based Pharmacokinetic Models for Tacrolimus

Despite the established history of PopPK models in tacrolimus dosing support, PBPK modeling adoption faces several challenges. The complexity of drug disposition mechanisms in transplantation and limitations of closed-code software packages necessitate more complex models, requiring flexible platforms and specialized expertise.

PBPK models for tacrolimus must address multiple factors contributing to patient variability. Critical considerations include low and variable bioavailability due to poor solubility, first-pass effects influenced by CYP3A5 and P-glycoprotein transport, and elimination pathways particularly relevant in transplant patients. Models must also account for hematocrit’s influence on blood-plasma partitioning and distribution across tissues, including liver, kidneys, adipose tissue, and blood cells.

Among PBPK modeling publications for tacrolimus, four significant studies focused on kidney transplantation. Emoto et al. developed a comprehensive Simcyp-based model using a middle-out approach [101]. Their work confirmed the impact of CYP3A4 abundance, hematocrit, and serum albumin levels on tacrolimus pharmacokinetics, though P-glycoprotein contributions were not considered. The model successfully explored pediatric populations, attributing age-dependent changes primarily to CYP3A ontogeny.

Prado-Velasco et al. advanced the field by investigating circadian modulation in pediatric patients using Phys-PK [102]. Their model, incorporating major organ compartments and demographic variables, demonstrated superior predictions compared to PopPK approaches. They applied Poulin and Theil methods for tissue-plasma partitioning [103], revealing significant intra-patient variability during formulation transitions.

A minimal PBPK model by Itohara et al. using Simcyp focused on absorption parameters [104], though it excluded critical factors like solubility and P-glycoprotein polymorphisms. Van der Veken et al. later addressed these limitations by incorporating mechanistic absorption modeling [105]. Their work revealed that amorphous solid dispersion causes tacrolimus to behave as a BCS class 1 rather than class 2 compound, suggesting absorption may not be the primary source of variability in exposure. Recent advances include El-Khateef et al.’s work combining therapeutic drug monitoring with PBPK modeling to investigate chronic kidney disease effects [106]. The approach has also expanded to other transplant types, including liver [107], lung [108], and heart [109], with applications extending to pregnancy populations [110].

PBPK modeling has emerged as a valuable tool for understanding tacrolimus pharmacokinetics across diverse populations and conditions. While these models demonstrate promise in optimizing dosing strategies and predicting drug interactions, external validation remains crucial for broader clinical implementation. These insights are particularly valuable for special populations, where personalized dosing strategies significantly impact therapeutic outcomes.

### Machine Learning

Machine learning (ML) is a branch of artificial intelligence (AI) that allows computers to learn and make predictions from data without being explicitly programmed to perform each task [111]. Instead of following pre-defined instructions, ML systems use algorithms that analyze data and look for patterns to improve their performance on specific tasks autonomously. The modeling steps consist of: i) data collection and clearing of data for inconsistencies, ii) selection of the best algorithm suitable for the specific purpose (supervised learning, unsupervised learning and reinforcement learning algorithms), iii) training of the model with training data to adjust parameters and learn, iv) performance evaluation of the model with unseen test data, v) optimization of parameters and model deployment in a real-world environment where it can adapt and improve with new data.

PBPK modeling approach offers the possibility of minimizing the animal studies and only using drug-related input parameters for PK predictions in humans. The evaluation of the prediction performance of different software packages as a function of data availability and software options, in a bottom-up approach, showed that predictions are not always within the acceptable range. Moreover, model prediction could not be improved with modeling strategies, but with unbiased parameters used to inform the model [111, 112]. ML is already available to generate unbiased and optimized parameters to be used in bottom-up PBPK modeling approach [113]. The top-down and middle-out approaches can also benefit from AI and ML. For example, AI can contribute to identifying all published PK data of the literature for a drug. Also, these approaches can contribute to optimizations of parameters in the middle-out approach such as tissue Kp values, specific enzyme intrinsic clearance values, or unbound fractions among others. Parameter optimization is particularly labour-intensive and typically not automated, relying heavily on the modeler’s expertise to identify the best-fit parameters. AI and ML could help in this process with ML algorithms. These technologies can test numerous combinations at a speed far beyond human capabilities. Therfore, AI could identify the optimal model configuration that best fits all available clinical data.

ML is still evolving, so that its contribution to advances in MIPD is still scarce. Few ML models have been developed for tacrolimus in renal transplantation with good predictions in both cases. Tang et al [114] used ML to predict stable dose in a large Chinese cohort (N = 1,045 recruited patients, 80% used for the derivation cohort and 20% used for the validation cohort). Among all the ML models, regression tree performed best in both derivation and validation cohorts. Covariates statistically significant in the derivation cohort were CYP3A5 genotype, hypertension and use of omeprazole. Sanchez-Herrero et al also applied ML to predict tacrolimus blood concentrations in a paediatric cohort of renal transplant patients (N = 21) [115]. The ExtraTrees Regressor algorithm had superior performance than the other algorithms tested. In both studies the authors reported acceptable values of metrics used to evaluate the accuracy of predictions. Woillard et al investigated whether ML models (Xgboost) accurately estimated tacrolimus AUC in transplant patients using sparse concentration data [116] and also explored the training of Xgboost ML models on simulated tacrolimus concentration-time profiles [117]. Xgboost machine learning models trained on simulated concentration-time profiles from literature PopPK models enable precise tacrolimus AUC estimation based on sparse concentration data. Further studies are still required to advance on the application of ML on MIPD.

### Other Tools for a More Efficient Modeling With NONMEM: ChatGPT and Gemini Large Language Models for Generating Initial Codes Templates of NONMEM

Shin et al evaluated the utility of the ChatGPT4.0 and Gemini Ultra 1.0 large language models for NONMEM coding tasks relevant to pharmacometrics and clinical pharmacology [118]. Their conclusions were that these tools could be useful in the earlier steps to obtain early versions of the codes, but that these codes still require careful checking for errors and improvements before implementation.

## Conclusion

In conclusion, understanding the predictive factors of variability in tacrolimus pharmacokinetics is essential for achieving precision dosing and optimizing therapeutic outcomes. Factors such as genetic polymorphisms (e.g., CYP3A5 expression), demographic characteristics, comorbidities, drug-drug interactions, and physiological changes significantly influence tacrolimus absorption, distribution, metabolism, and clearance. Recognizing these variables allows for more accurate dose adjustments, reducing the risk of underdosing or overdosing and minimizing associated adverse effects or graft rejection.

The integration of these predictive factors into MIPD frameworks, supported by advanced PopPK models and decision-support software, enables individualized treatment strategies tailored to each patient’s unique profile. This approach not only enhances the safety and efficacy of tacrolimus therapy but also underscores the importance of personalized medicine in improving outcomes for vulnerable populations, including pediatric, elderly, and critically patients.

MIPD is endorsed by tacrolimus PopPK modelling of tacrolimus. Population and PBPK models, together with individualized adjustment tools such as Bayesian prediction, allow for more accurate drug management. However, challenges such as high variability and integration of complex clinical covariates remain. Future research aims to integrate more detailed physiological models and pharmacogenetic approaches to further optimize therapy. None of these approaches replace the others, rather they complement each other.

Despite the promise of MIPD in optimizing therapeutic drug monitoring, several hurdles must be addressed to facilitate its implementation in clinical practice. Key challenges include limited availability of robust data for model validation, unclear regulatory pathways for endorsing MIPD tools, and the high costs associated with software licenses and training healthcare professionals. Additionally, the complexity of MIPD models and tools can hinder their practical use, requiring user-friendly interfaces and continuous updates to maintain relevance and accuracy. Prospective clinical studies demonstrating improved outcomes, such as reduced toxicity or enhanced efficacy, would be valuable. Furthermore, collaborative efforts involving diverse stakeholders -such as researchers, clinicians, regulators, and patient groups- could support model validation and integration into routine care. Education and training programs tailored to healthcare providers will enhance trust and adoption of MIPD approaches. By addressing these challenges through targeted studies and multistakeholder collaboration, the widespread implementation of MIPD can become feasible and impactful.
